# Myocilin mutations among primary open angle glaucoma patients of Kanyakumari district, South India

**Published:** 2007-04-02

**Authors:** Rajiv Rose, Muthusamy Karthikeyan, Balakrishnan Anandan, Gopalswamy Jayaraman

**Affiliations:** Department of Genetics, Dr. ALM PGIBMS, University of Madras, Taramani Campus, Chennai, Tamil Nadu, India

## Abstract

**Purpose:**

Glaucoma can be defined as optic neuropathy leading to irreversible blindness if not treated in time. Primary open angle glaucoma (POAG) is the most common form of glaucoma. The myocilin (*MYOC*) gene has been found to mutate in both sporadic and familial cases of POAG worldwide. About 90% of these mutations have been seen to cluster at exon III of the gene. There are documented reports of mutations in the *MYOC* gene among POAG patients from different parts of India. The southernmost tip of the Indian subcontinent (Kanyakumari district) has remained isolated from all these studies. The aim of this study was to indicate or rule out the disease causative role of the *MYOC* gene mutations in these patients by screening the *MYOC* gene for mutations among POAG patients of the Kanyakumari district.

**Methods:**

One hundred POAG patients from the Kanyakumari District of South India were recruited for the study. The *MYOC* gene was screened using the PCR-SSCP methodology followed by DNA sequencing. The sequences were analyzed using BLAST. Secondary structures of the amino acid sequences with a variation were predicted.

**Results:**

Two probable disease-causing variations (mutations), Ser331Thr and Pro370Leu, were each observed in one patient apiece. Two polymorphisms, (Tyr347Tyr and Thr325Thr) were also observed in the patients. Ser331Thr is a novel conservative change while Pro370Leu is a widely reported mutation with an associated severe disease phenotype.

**Conclusions:**

The presence of the mutations in the patients suggests the causative role of the *MYOC* gene among POAG patients in the Kanyakumari district of India. The mutation frequency of 2% corresponds well with the other reports from India and other countries. However, the mutation rate reported from a population in the eastern part of India was much higher. Screening of patients from different parts of India is essential to estimate the overall mutation frequency. More functional studies on the *MYOC* gene are required to elucidate the pathophysiology of POAG.

## Introduction

Glaucoma is a term used to describe a group of disorders that have optic neuropathy as a common feature. Optic neuropathy is optic nerve head cupping or degeneration of the optic nerve. This cupping of the optic nerve head initially leads to the loss of peripheral vision and, if not treated in time, results in irreversible blindness [1]. Among the different forms of glaucoma, the primary open angle glaucoma (POAG) is the most common type [2,3]. There are two forms of POAG: adult onset and juvenile onset. These are mainly distinguished based on the age of onset of the disease. However, juvenile onset POAG usually has a more severe disease phenotype compared to the adult onset type [2,4].

India has a high prevalence of glaucoma with POAG being the most common form of glaucoma [5]. In the WHO report on the prevalence of glaucoma in India, 1% of the population was stated to be blind of which 12.8% was due to glaucoma [6]. The prevalence of POAG in South Indian populations has been reported to be 1.62% [7,8]. This prevalence rate is similar to that observed in Western populations.

A family history of glaucoma has long been recognized as an important risk factor for the disease. The common adult onset POAG is inherited as a complex trait, while the juvenile onset POAG exhibits autosomal dominant mode of inheritance [9]. The prevalence of POAG among first degree relatives of affected patients is as high as 16-22% [10,11]. Thus far, 22 gene loci have been linked to POAG [9,12-26].

Three genes, *MYOC*, *OPTN*, and *WDR 36*, have been found to mutate among POAG patients [23,27,28]. The *MYOC* gene, located on the GLC1A locus, was initially known as the trabecular meshwork-inducible glucocorticoid response (*TIGR*) gene. *MYOC* sequence variations associated with POAG were first observed by Stone et al. [27]. Mutations in the *MYOC* gene have now been reported to be present among POAG patients from almost all parts of the world [1,29-41]. An average mutation frequency of 1.4-4.6% has been observed among POAG patients worldwide [29,32,35,42]. The *MYOC* gene consists of three exons with two intervening introns. Exon III is the largest of the three and is referred to as the olfactomedin-like domain due to its homology to olfactomedin. More than 90% of the mutations reported so far are clustered to exon III of the gene [34,43].

There are three reports currently available on populations from different parts of India that estimate the mutation frequency among randomly selected, unrelated POAG patients. Sripriya et al. [40] reported a mutation frequency of 2% among 100 patients screened from Chennai (South India). Kanagavalli et al. [39] found a mutation frequency of 2% among 107 POAG patients screened from Madurai (South India). While Mukhopadhyay et al. [36] reported mutations among 4 of 56 POAG patients (about 7.1%) screened from Kolkata (East India).

There are two major reasons to screen a gene for mutations: (a) to implicate the gene as a cause of a particular disease; and (b) to identify as many mutations as possible for the purpose of understanding genotype-phenotype relationships, comparing mutation profiles in different populations or constructing practical genetic tests for clinical use [34]. The present study was aimed to implicate or rule out the involvement of mutations in the MYOC gene in disease causation among POAG patients of the Kanyakumari district of South India. This region has never been included in any such genetic or epidemiological study pertaining to POAG. This study would serve as an initiator for further POAG research.

## Methods

### Clinical Samples

This study recruited 100 patients from hospitals in the Kanyakumari District. All patients had been given the diagnosis of adult/juvenile-onset POAG. Eighty-seven of these were adult onset cases, and 13 were patients with juvenile onset open angle glaucoma. Twenty-five patients had at least one family member affected by POAG. In three cases, the family history could not be confirmed. An equal number of age- and sex-matched controls from the same district were included in the study. Informed consent was obtained from each individual before blood was drawn. Each patient underwent a complete ophthalmic examination, which consisted of measurement of intraocular pressure (IOP), gonioscopic evaluation of the angle, examination and documentation of the optic disc, and visual field testing. The criteria for diagnosing glaucoma, based on previous reports [4], were that the patient had been (1) treated for glaucoma prior to their involvement in the study or (2) had at least two of these features: (a) an IOP of greater than or equal to 22 mmHg, (b) an increased optic cup to disc ratio (where the normal value is 0.3, and glaucomatous cupping is greater than or equal to 0.5), and (c) visual field abnormalities with no other apparent cause for the loss in vision.

Classification of adult and the juvenile onset POAG was based on age of disease onset; adult onset was considered to be after 40 years of age, whereas juvenile onset was before this age [4,9].

### Analysis of the *MYOC* gene

Genomic DNA was isolated from the blood samples of patients and controls using the salting out method of Miller et al. [44]. Eight primer sets were designed to amplify the gene in fragments of <300 bp (Table 1) so as to enable analysis of the amplicons using the single strand conformation polymorphism methodology. PCR amplifications were carried out in 20 μl reaction volumes containing about 100 ng of genomic DNA, 5 pmol of each primer (forward and reverse), 2 mM of each dNTP, 0.5 U of Taq polymerase with a standard buffer containing 1.5 mM MgCl_2_. Amplification was carried out under the following conditions: initial denaturation 94 °C for 5 min, followed by 30 cycles of denaturation 94 °C for 30 s, annealing for 30 s (for each annealing temperature [Ta], please Refer to Table 1), extension -72 °C for 30 s, followed by a final extension at 72 °C for 2 min.

**Table 1:** PCR amplification conditions.

Primer name	Sequence	Forward/ Reverse	Binding Site#	Ta*	Product Size
RR3	5' TggATTAAgTggTgCTTC 3' - 18mer	Forward	c.731-40 to c.731-23 (intron II)	58 °C	227 bp
RR4	5' TggCTgATgAggTCATAC 3' - 18mer	Reverse	c.900toc.917 (exon III)		
RR5	5' ggATgTCCgCCAggTTT 3' - 17mer	Forward	c.879 to c.895 (exon III)	60 °C	260 bp
RR6	5' CAATgTCCgTgTAgCCAC 3' - 18mer	Reverse	c.1121 to c.1138 (exon III)		
RR7	5' TggCTACCACggACAgTT 3' - 18mer	Forward	c. 1089 to c.1106 (exon III)	62 °C	243 bp
RR8	5' gAgTgTAgCTgCTgAC 3' - 17mer	Reverse	c.l315 to c.1331 (exon III)		
RR9	5' CCTTCATCATCTgTggCA 3' - 18mer	Forward	c.l286 to c.1303 (exon III)	64 °C	248 bp
RR10	5' gTACAgCTTggAggCTT 3' - 17mer	Reverse	*5to *21 (3'UTR)		
RR1	5' AgAgC TTTCCAgAggAAg 3' - 18mer	Forward	-36 to -19 (5'UTR)	64 °C	248 bp
RR2	5' ATgACTgACATggCCTgg 3' - 18mer	Reverse	c.195 to c.212 (exon I)		
RR11	5' GTCCCAATgAATCCAgCT 3'- 18mer	Forward	c.l64 to c.181 (exon I)	62 °C	268 bp
RR12	5' TTgCTgTAggCAgTCTCC 3' - 18mer	Reverse	c.414 to c.431 (exon I)		
RR13	5' gACCAgCTggAAACCCA 3' - 17mer	Forward	c.385 to c.401 (exon I)	60 °C	256 bp
RR14	5' TgCTgAACTCAgAgTCC 3' - 17mer	Reverse	c. 604 +20 to c.604 +36 (intron I)		
RR15	5' CATAgTCAATCCTTgggC 3' - 18mer	Forward	c.605 -61 to c.605 -44 (intron I)	64 °C	231 bp
RR16	5' TAAAgACCACgTgggCAC 3' - 18mer	Reverse	c.730 +27 to c.730 +44 (intron II)		

The following are given for each primer set: primer name, sequence, binding site, annealing temperature, and expected amplicon size for each primer set. The asterisk indicates the annealing temperature (Ta).

The amplicons were mixed with the loading dye (bromophenol blue, xylene cyanol, formamide, and Na_2_EDTA) and double distilled water in the following ratio: 2 μl amplicon, 2 μl dye, and 16 μl double distilled water. The combination was heat denatured for 5 min in boiling water and electrophoresed on a composite (acrylamide, bis acrylamide, and agarose) gel [45] for 13 to 14 h. The percentage of the gels used and the electrophoresis voltage varied for amplicons of the different primers sets. Gels were silver stained as per the protocol of Bassam et al. [46]. The amplicons with a mobility shift were reamplified, column purified, and sequenced using dye termination chemistry and read using the 96 capillary 3730x1 DNA analyzer (Applied Biosystems, Foster City, CA). Sequences were analyzed using BLAST to identify the variation(s).

DNA sequences with a variation were translated in silico to obtain the corresponding amino acid sequences. The amino acid sequences were subjected to the secondary structure prediction using the Garnier-Osguthorpe-Robson (GOR) prediction method [47,48], a means of predicting the secondary structure of proteins. This method forecasts the secondary structure of a sequence by calculating the probability for each of the four structure classes (helix, sheet, turn, and loop) based on the central residue and its neighbors from the precalculated matrices. The highest is then selected for each residue.

## Results

**Figure 1:**
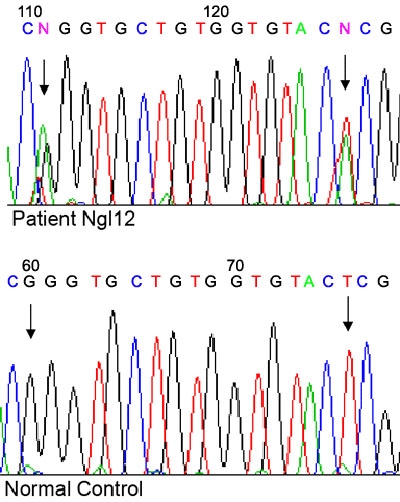
Heterozygous Ser331Thr mutation and Thr325Thr polymorphism in the *MYOC* gene. Chromatogram sequence derived from patient Ngl 12 with the T>A transversion and G>A transition (indicated by arrows) compared to the normal control.

**Figure 2:**
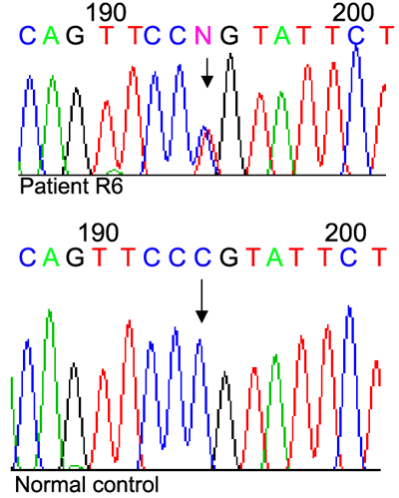
Heterozygous Pro370Leu mutation in the *MYOC* gene. Comparison of chromatogram sequences derived from patient R6 and normal control. Arrow marks the C>T transition compared to the normal control.

Four different types of sequence variations were obtained. Two of these were nonsynonymous mutations i.e., the variations were both in the nucleotide sequence, and the corresponding amino acid sequence (missense mutation), namely Ser331Thr and Pro370Leu, showed up in at least one patient (Figure 1 and Figure 2). These were probable disease-causing variations. The other two were synonymous variations, meaning the changes were in the nucleotide sequence alone and not in the corresponding amino acid sequence: (1) Tyr347Tyr in four patients and (2) Thr325Thr in one patient. All sequence variations were observed in the heterozygous state and only in the patients' samples.

The Ser331Thr mutation (T>A transversion at nucleotide 991) was observed in one patient, who also had the Thr325Thr synonymous mutation (G>A transition at nucleotide 975). This patient was 68 years old at the time of sample collection. At the time of blood drawing the patient's IOP was 20.6 mmHg OD and 20.6 mmHg OS. The patient was was being treated with 0.5% levobunolol for POAG. The cup to disc ratio revealed a totally excavated disc in the left eye and was 0.9 in the right eye. The left eye was totally blind with only light perception, and the right eye had about 25° of vision. The patient was hypertensive (180/100 mmHg). The patient denied a family history of the disease.

The Pro370Leu (C>T transition at nucleotide 1109) was the other mutation observed in one patient only. The patient was 57 years old at the time of sample collection and she had been affected by glaucoma at about 16 years of age. During the last stages of her vision, she had highly elevated pressures (readings not available), open angles, and no other systemic or ocular abnormalities that could have resulted in the loss of vision, indicating POAG. This is a typical example of a rapid progressive JOAG with an early age of onset. It was later learned she had a family history of glaucoma. Her brother developed JOAG before the age of 30 and six of her uncles (distant relatives) developed either POAG or primary angle closure glaucoma (PACG). Her brother with POAG, three of her uncles (first cousins once removed), and one second cousin who had POAG/PACG were also included in the present study. However, none of them had any variation in the MYOC gene.

The other synonymous variation or polymorphism was the Tyr347Tyr (1041 T>C) change. This was seen in four patients.

Random sequencing of five patients and five normal control samples of each primer set confirmed the negative results of SSCP analysis.

## Discussion

This is the first such study from this part of India. The patients from the Kanyakumari district had two mutations: (1) Pro370Leu (1109 C>T) and (2) Ser331Thr (991 T>A). The Ser331Thr is a novel mutation with no report of it available at present as far as our literature search showed. The other mutation has been reported in other populations worldwide.

**Figure 3:**
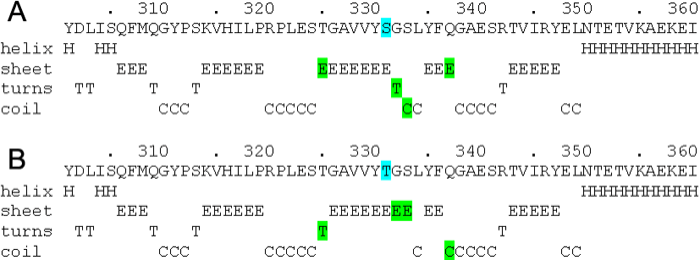
Secondary structure prediction of the mutant sequence (Ser331Thr) using the GOR method. Comparison between the predicted secondary structures of control (**A**) amino acid sequences of myocilin and the amino acid sequences of myocilin with the Ser331Thr variant (**B**; only the region where there was a change in the predicted secondary structure has been shown).

The Ser331Thr mutation observed in this study is a conservative change of serine to threonine. However its absence in the controls and the effect of this change on the predicted secondary structure of the protein (Figure 3) suggests that it might have a causal effect. Alhough there is no direct evidence of this being a causal factor of glaucoma, the benign nature of this variant cannot be assumed. The fact that this change is not mentioned in the literature and that it was absent in the normal controls indicates this is a mutation or a probable disease-causing variation.

An earlier report mentions that though the nonconservative changes were presumably more injurious to the functioning of the myocilin protein than conservative changes, the conservative changes have also been classified as probable disease-causing mutations if they were absent in the controls [34].

The Pro370Leu alteration was observed in one patient, who had a severe disease phenotype with an early age of onset of the disease (diagnosed at 16 years), rapid progression of the disease, and a positive family history of the disease. The severe nature of the disease phenotype associated with this variant correlates well with earlier reports [30,33,49]. The proband reported by Damji et al. [50] had a similar clinical history as that of the aforedescribed patient: early age of onset, much before sample collection, lack of initial ophthalmological examination records, and a family history of the disease.

This mutation has also been reported in an Indian patient from Kolkata (eastern part of India) [36]. The patient had pressures of 24 mmHg OD and 32 mmHg OS and cup to disc ratios of 0.8 OD 0.7 OS. Vision tests revealed presence of an arcuate scotoma in the superior and inferior halves with nasal steps OD and a scotomatous defect in the superonasal quadrant OS.

The occurrence of this mutation in patients of varying ethnicity (Indian, English, French, North American, Japanese, and German populations) suggests this is a prevalent mutant allele of MYOC. The severe nature of the disease in patients with this mutation indicates that the loss of proline at this position may acutely affect the normal function of the protein [32]. However, further studies are needed to correlate disease severity with this mutation.

The change C>T transition at nucleotide 1109, results in a change from proline (a polar amino acid) to a hydrophobic leucine molecule. This transition occurs in the CpG dinucleotide. The importance of CpGs in the disease causation was suggested by Mukhopodhyay, et al. [36].

The translationally silent polymorphism in codon 347 has a relatively high incidence in subjects from the Kanyakumari district (about 4%). These observations indicate this is a frequent polymorphism found among POAG patients of this part of India.

Both the mutations presented here have been predicted to affect the myocilin protein's secondary structure. Secondary structure prediction of the Ser331Thr mutant amino acid sequence by GOR revealed a removal of turn "T" at the amino acid 332 followed by an addition of a β sheet "E" at amino acid 332 and 333. A substitution of β sheet "E" to turn "T" at amino acid 325 was predicted and β sheet "E" was expected to coil "C" at 337. The Pro370Leu results in a change from a polar amino acid to a hydrophobic amino acid. This mutation also results in changes in the predicted secondary structure. Moreover, it has been elucidated that proline has a side chain that inhibits (α helix) formation and fits poorly in the α helix conformation, while leucine is one of the good α helix formers [51]. Since the function of the *TIGR/MYOC* gene has not yet been fully elucidated, the effects of single amino acid changes cannot be precisely predicted and can only be hypothesized [52].

The findings suggest that the *MYOC* gene is involved in the causation of POAG in this part of India as well. The mutation rate was 2% among POAG patients of the Kanyakumari district This is not a high rate, but the frequency coincides with that reported from other parts of India and the world.

A larger data set including more patients and functional studies on the *MYOC* gene are required to elucidate the pathophysiology of POAG.
